# The higher they go the harder they could fall: The impact of risk-glorifying commercials on risk behavior

**DOI:** 10.1371/journal.pone.0225884

**Published:** 2019-12-03

**Authors:** David F. Urschler, Hanna Heinrich, Stefanie Hechler, Peter Fischer, Thomas Kessler

**Affiliations:** 1 Department of Social Psychology, Institute for Psychology, University of Jena, Jena, Germany; 2 Department of Social and Organizational Psychology, Institute for Psychology, University of Regensburg, Regensburg, Germany; Middlesex University, UNITED KINGDOM

## Abstract

Previous research on risk-glorifying media has provided encompassing evidence for a positive connection between risk-glorifying contents and (a) risk-positive emotions, (b) risk-positive cognitions and attitudes, and (c) risk-positive behavioral inclinations. Nevertheless, little evidence shows whether risk-glorifying content increases actual risk behavior. We conducted three experimental studies to assess whether risk-glorifying commercials increase risk behavior. In all studies, participants were randomly assigned to a risk-glorifying or a neutral commercial. Additionally, in Study 2 participants were randomly assigned to an arousal or a non-arousal condition to test the mediating effect of arousal. In Study 3, we tested the mediating effect of the accessibility to risk-positive cognitions. We measured participants’ risk behavior via the risk assessment ramp (RAR). Our results revealed that participants who watched the risk-glorifying commercial walked faster to the jumping-off point (Studies 1, 2, & 3) and would have jumped from a higher level (Studies 2 & 3), thus, indicating the exposure to risk-glorifying media content increases people’s risk behavior. Neither arousal nor the accessibility to risk-positive cognitions mediated the effect of risk-glorifying media content. Beyond our findings, we offer a new tool to assess risk behavior that is effective and easy to apply.

## Introduction

Many people are attracted to extreme sports events, which take place at well-known places all over the world. For instance, up to 30,000 fans cheer for freestyle motocross riders at the Plaza de Toros Monumental (Mexico City, Mexico) who perform spectacular stunts with characteristic names such as “kiss of death backflip”. An off-road motocross race true to the motto “tougher than iron and harder than steel” attracts every year 20,000 people. In this race (the allegedly hardest off-road race in the world), only nine out of 500 starters were able to finish in 2016. The winner merely receives a piece of rock as a trophy, gains recognition of their comrades, and is admired by their fans.

Besides that tens of thousands of fans cheer for their admired athletes, several companies use the footage of those events to produce spectacular commercials (including stunts that went badly wrong). Here the question arises of how people react to those commercials that reward engaging in risk behavior (for a definition and overview see [1]), especially, since risk-taking behavior is one of the main causes of lethal injuries among children, adolescents, and young adults [2].

Previous research showed that commercials could foster various types of risk behavior, for reviews see [3,4]. For example, previous studies provide evidence for a link between alcohol-promoting commercials and the uptake of drinking among non-drinking adolescents, and an increased consumption among drinking peers [5–7]. This effect can be observed in cross-sectional and longitudinal studies [5]. Despite the compelling empirical and meta-analytical evidence for a link between alcohol-promoting commercials and subsequent alcohol consumption, previous experimental research [8–10] and a review [11] has also revealed mixed findings. Additionally, previous experimental research has also revealed negative effects [12,13]. In a similar vein, exposure to tobacco commercials is associated with an increased likelihood that young people will start to smoke [14]. However, the link between increased risk behavior after exposure to a commercial (i.e., alcohol consumption) and additional risk behaviors (i.e., driving under the influence of alcohol) remains somewhat unclear [7].

Several sad anecdotes underpin the assumption that risk-glorifying media content is a key predictor for risk behavior in society. For example, a 24-year-old-man tried to copy a stunt (i.e., racing downhill with a shopping cart) that had been broadcasted on MTV’s famous series “Jackass”. The attempt resulted in the young man’s death. Another “Jackass” copycat severely burned himself while replicating a broadcasted stunt [3]. Moreover, illegal street racers caused a fatal car crash in Toronto, Canada, killing an innocent taxi driver. In one of the racers’ vehicles officials found a copy of a street racing videogame that glorifies and simulates, reckless driving and illegal street racing [15]. An advertisement for this game reads as follows, “No tracks and no simulation. It's about taking your ride to the limit and beyond, nailing perfect 200 mile-an-hour drifts, slamming your friends off the road, outsmarting the cops and getting away with it in style”. Following these sad anecdotes, an investigation of the effect of risk-glorifying commercials is of high societal interest, although previous experimental research also revealed that the exposure to a risky driving motor vehicle commercial had no immediate effect on risk-positive attitudes, emotions or risky driving inclinations[16].

### Effects of risk-glorifying media content

Previous research on the effects of risk-glorifying media content (for a review see [4] and a meta-analysis see [3]), provided encompassing evidence that risk-glorifying media content leads to risk-positive emotions, risk-positive cognitions and attitudes, and risk behavior. This effect could be observed for different types of media (i.e., commercials, music, movies, and video games) and different types of risk-taking behavior [3]. Moreover, previous research revealed that risk-glorifying media content increases the accessibility of risk-promoting cognitions, which in turn results in increased risk-taking inclinations [17]. To be more specific, adolescents who prefer music with risk-glorifying lyrics are more likely to engage in risky sexual behavior than their contemporaries who prefer music with neutral lyrics (e.g., [18,19]).

Concerning movies, previous research showed that teenagers who watched movies in which alcohol consumption was positively depicted had an increased risk for trying drinking and binge drinking in the future [20]. A consumption of those movies was positively connected with an intention to drink [21]. Relatedly, people who watched movies that presented smoking characters increased smoking initiation compared to those who did not view those movies [22–24].

With regard to video games, a series studies revealed that playing racing games is associated with tailgating and competitive road traffic behavior, an increased number of reported accidents, and reduced cautious road traffic behavior [25]. Moreover, people who were playing racing games showed a higher accessibility to risk-glorifying cognitions and riskier driving behavior in simulated critical road traffic situations (this effect was only observed for male participants) than those who played a neutral game [25]. Additionally, 24 hours after playing a racing game (i.e., racing games that make players actively break rules), people showed an increased level of risk taking inclination in a driving simulator task in comparison to people who played a neutral game [26].

Concerning advertisements on TV, previous research revealed that exposure to alcohol advertisements on TV is positively correlated with an increased risk of alcohol consumption [27,28]. Similar patterns could be observed for the impact of tobacco advertisements on smoking [29,30], and for gambling advertisements on gambling attitudes and behaviors [31].

## The present research

Although previous research has provided great insight into questions regarding the consequences of being exposed to risk-glorifying media (i.e., commercials), surprisingly, little is known whether exposure to risk-glorifying contents increases actual risk behavior. Precisely, previous experimental research mainly measured (a) accessibility to risk-glorifying cognitions [17,32,33], or (b) assessed risk-taking behavior under the usage of simulated critical road traffic situations [16,26]. Most studies that assessed risk behavior (i.e., smoking, pathological gambling, drug abuse) were correlational in nature, and cannot explain a causal relationship [29,34–38]. Note that previous experimental research that investigated the impact of risk-glorifying content mainly assessed alcohol consumption [10,12,39].

Therefore, the present research aims to extend the existing literature by testing the impact of risk-glorifying commercials on risk-taking behavior in a different setting. Additionally, previous research showed that the convergent validity between propensity measures (e.g., risk-positive emotions, risk-positive cognitions and attitudes) and behavioral measures (e.g., gambling behavior, risky-decisions) is relatively weak. Hence, it is important to close this gap, cf. [40,41]. Closing this gap can enhance the generalizability of media effects (i.e., commercials) of risk behavior in experimental settings. Furthermore, closing this gap can lead to an improved theoretical understanding of how media effects are transferred (i.e., via arousal and via accessibility to risk-positive cognitions) to actual risk behavior. To the best of our knowledge, this is the first attempt to investigate the impact of risk-glorifying media content (i.e., commercials) on actual risk behavior.

We base our predictions on socio-cognitive models that incorporate social learning, [42,43], social information processing [44], the social-cognitive model of media violence [45], the excitation transfer model [46], and the cognitive neoassociationist model [47]. Given that cognitions (for a definition see [48]), emotions, and behavioral scripts are assumed to be organized as neuronal networks in the cognitive system, e.g., [49], these models explain media effects via activation of these networks. Media content triggers ideas, which prime other, semantically related thoughts and cognitions in turn [47]. For example, risk-glorifying media content activate the concept of aggression and thus increase the cognitive accessibility of cognitions, emotions, and behavioral scripts related to it [3,17,25]. This way of arguing is also in line with the predictions of the general learning model [50]. Consequently, we hypothesize that people who were exposed to a risk-glorifying commercial behave riskier than those who watched a neutral commercial.

## Pretest

We pretested our materials (i.e., commercials) to check whether people perceive the risk-glorifying commercial as more risk-glorifying than the neutral commercial. Additionally, we wanted to rule out that participants’ risk behavior is affected by the commercials’ overall evaluation (e.g., liking, interest). Given that previous research successfully used extreme sport pictures as risk-glorifying stimuli [25], we hypothesize that participants evaluate the risk-glorifying commercial’s content as riskier than the neutral commercial’s content. Note that we report all measures, manipulations, and exclusion in our studies. Additionally, all materials, questionnaires, data, and R-scripts are available on the OSF platform (osf.io/rq6yj).

### Method

We confirm that the reported research is in accordance with the APA’s ethical standards and were performed in line with the Declaration of Helsinki. The following studies had been approved by the University of Regensburg’s ethics committee before they were conducted. All participants provided oral (Pretest) or written (Studies 1, 2, & 3) informed consent for their participation.

#### Participants and design

Twenty-four students (16 female) evaluated the material. Their age ranged from 19 to 26 years (*M* = 21.83, *SD* = 1.95). Participants were recruited via postings on the hallways at the psychology department at a University. Oral informed consent was obtained from all participants before they were randomly assigned to either the risk-glorifying condition or the neutral condition. Participants received no payment for participation. The pretest was based on a one factorial between-subjects design (“risk-glorifying commercial” vs. “neutral commercial”). In all studies (pretest and Studies 1 to 3), participants and experimenter were blind to the experimental conditions (double-blind).

#### Procedure

Participants were informed that they are participating in a study about commercial perception. They watched either the risk-glorifying or the neutral commercial in groups of three to four people in a seminar room (for a detailed description of the commercials see material section). After participants had watched the commercial, they completed a short questionnaire to assess overall evaluation and risk perception (see dependent variables).

#### Material

Participants were exposed either to a commercial of a sporting goods company (neutral condition) or to a commercial of an energy drink company (risk-glorifying condition). Both commercials had a duration of approximately four minutes.

In the *neutral* condition the commercial depicted several soccer matches of a tournament which took place in an iron cage on a discarded cargo ship. Each participating team in the commercial consisted of three very well-known international soccer players who performed a number of spectacular ball tricks during the matches. Previous research had successfully used soccer games as neutral stimuli [25]. Therefore, we believe that the sporting goods commercial is appropriate as a neutral stimulus.

In the *risk-glorifying* condition the commercial portrayed different extreme sports (e.g., freestyle motocross, snowboard freeriding, skateboarding, mountain bike downhill racing, and big wave surfing). Participants who watched the risk-glorifying commercial observed several stunts connected to a specific extreme sport such as backflips with motocross bikes. Importantly, not all of the portrayed stunts went well. For example, participants in this condition also watched motocross racers crashing badly. However, the majority portrayed positive outcomes.

#### Dependent variables

We asked the participants to evaluate the commercials based on the following question: “How much do the following statements fit the portrayed commercial?” Participants evaluated the commercials’ risk content on the following dimension: Appetite for risk, Freedom, Fear, Dangerousness, Thrill, Excitement, Adventure, Boldness, Safety (reverse coded), and Hazard (α = .83; all presented α-values are Cronbach α-values).

Participants rated the commercials’ overall evaluations (α = .77) on the following dimensions: I am able to remember the commercial well; The commercial bores me (reverse coded); The commercial is creative; The product is paramount; I got the message; I’m able to memorize the content well; The commercials is entertaining; and, The commercial gained my interest about the product. All dependent variables were assessed on a Likert scale ranging from 1 (*not at all*) to 7 (*absolutely*).

### Results and discussion

A *t*-test revealed that participants who watched the risk-glorifying commercial evaluated the content as riskier (*M* = 5.33, *SD* = 0.70) than those who were exposed to the neutral commercial (*M* = 4.59, *SD* = 0.77), *t*(22) = 3.15, *p* = .005, *d* = 1.28. With regard to overall evaluations, a *t*-test revealed that participants evaluated the neutral commercial (*M* = 4.43, *SD* = 0.85) and the risk-glorifying one (*M* = 4.09, *SD* = 1.19) not significantly different, *t*(22) = 0.79, *p* = .438, *d* = 0.32.

Taken together, the pretest’s results showed that the risk-glorifying commercial portrays riskier content than the neutral one, but on the other hand, the commercials did not significantly differ in their overall evaluation (i.e., positivity, interest etc.).

## Study 1

Study 1 aimed to investigate the effect of commercials’ content on risk behavior. Especially, we address the question whether watching a risk-glorifying commercial lead to increased risk behavior. We hypothesize that people who watch a risk-glorifying commercial show increased risk behavior.

### Method

#### Power analysis, participants, exclusion criteria, and design

Comparable previous research revealed large effects (Cohen’s *d*’s > 0.8; cf. [17]). To achieve sufficient statistical power (> .80) to detect large effects [51], we aimed for data from 52 participants. We factually collected data from 52 participants (46 female) with an average age of *M* = 19.90, *SD* = 2.03 and a range from 18 to 25 years. Participants were recruited via postings on the hallways at the psychology department at a University. In exchange for their participation, they received course credit, a cup of tea or coffee, and chocolate bars.

We excluded participants who were not convinced that they would have to jump-off the risk assessment ramp (RAR). Participants were randomly assigned to one of the two experimental conditions. This study was based on a one-factorial between-subjects design (type of commercial: “risk-glorifying” vs. “neutral”).

#### Procedure

**Fig 1:**
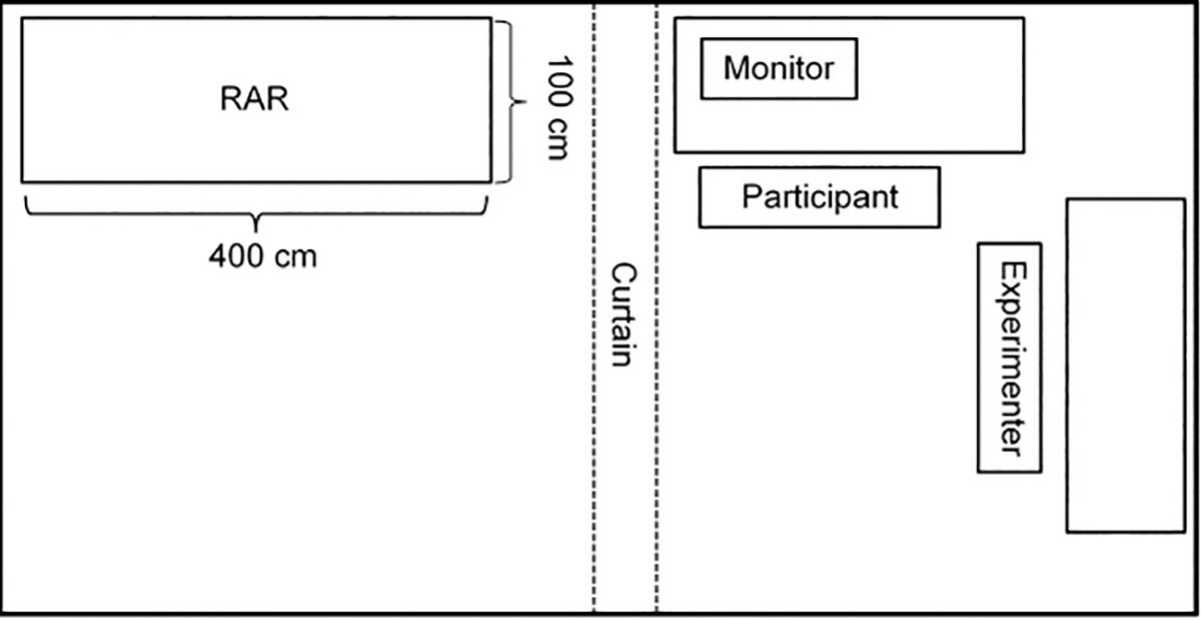
Laboratory setting.

We informed the participants that they would participate in two independent studies. They learned that the first study would deal with the attractiveness of different commercials and that the second study is about spatial perception. At the beginning, participants filled out a demographic questionnaire to assess age, gender, and citizenship. Subsequently, participants watched the commercial and rated the commercial on the eight “overall evaluation” items of the pretest (α = .82). We did not assess risk perception as this could have revealed the purpose of the study and led to demand effects. After finishing the rating, participants were thanked for participating in the first study and informed that they would be fully debriefed after the second study to save time (for an overview of the lab setting see Fig 1).

**Fig 2:**
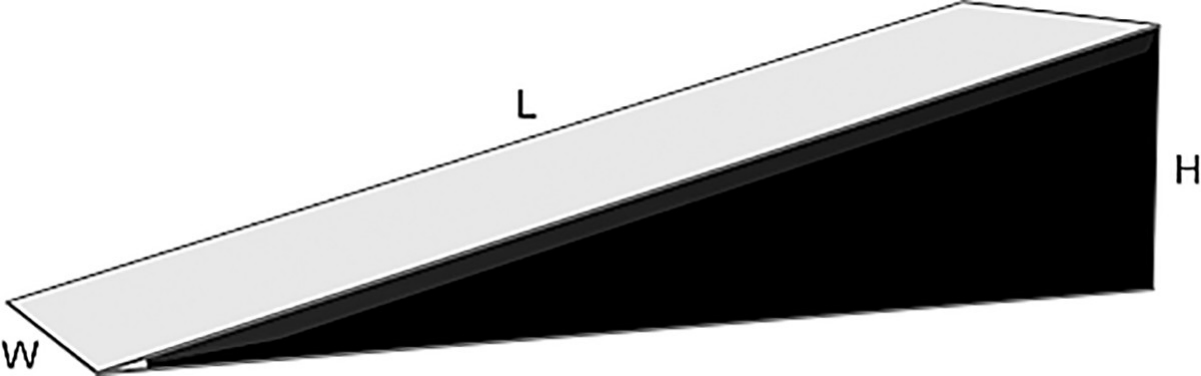
Symbolic illustration of the risk assessment ramp (RAR). L = length = 400 cm, W = width = 100 cm, H = height = 100 cm, gradient = 14°.

We informed participants that the second study’s alleged aim is to investigate people’s spatial perception skills when they are blindfolded. Participants were blindfolded to minimize participants’ visual information, which increases participants’ uncertainty as to whether they should jump or not [52]. Moreover, higher uncertainty increases a person’s physical risk of harming him- or herself [53]. The experimenter presented a model of a skewed wooden ramp of the RAR (for exact dimensions see Fig 2). Participants were informed that they will be blindfolded, will walk sideways up the RAR, and can stop once they reach the point from which they want to jump. Before blindfolding the participants, we asked them if they are acrophobic; if not, they were blindfolded and led to that RAR’s starting point. For each participant, the experimenter recorded the time (with a common stopwatch), starting when the participant began to walk until he or she reached his or her jump-off point. When they reached the jump-off point, the experimenter stopped the time (seconds and tenths of a second), and assessed their travelled distance (meters) and jump-off height (cm). The experimenter marked the outside edge of the upper foot on the RAR to assess the travelled distance and jump-off height (with a common measuring tape). Subsequently, participants were instructed to walk non-blindfolded off the RAR. No feedback was given while they were walking up the RAR, except a slight knock against the participant’s knee if he or she had come to close to the edge.

Subsequently, participants filled out a short questionnaire to assess whether they had already participated in a similar study, whether they were convinced that they had to jump, and their knowledge about the study’s purpose. Finally, the experimenter fully debriefed the participants according to APA ethical standards, handed them over their compensation for participating, and dismissed them.

#### Dependent variables

Based on time and distance measurements, we computed our first dependent variable walking speed, measured in meter per seconds (m/s). As a second dependent variable, we assessed participants’ jump-off levels, measured as height in cm. We believe that our dependent variables are suitable to assess risk behavior for the following reasons. Note that our participants were blindfolded.

Beyond that, previous research revealed that people with a reduced vision (our participants were blindfolded) adopt a more cautious walking strategy, which manifests by a decreased walking speed [54,55]. Conversely, this means that an increased walking speed indicates less cautious behavior. This argument is nuanced by the finding that walking (without vision impairment) on inclined surfaces, such as the RAR, decreases gait stability [56], which, in turn is an indicator for an increased risk of falling [57]. Thus, we argue that walking speed assesses risk behavior appropriately.

In line with previous research, jumping off an increased level is a type of risk behavior, in which people often engage [58–60]. Moreover, people who would be willing to jump off an increased level have an increased risk of injuring themselves [61]. Finally, reduced vision increases the risk of falling on a plane surface [62] and given that, our participants were blindfolded, we argue that walking speed and jump-off levels are indicators of risk behavior.

### Results

#### Check for interfering effects and suspicion

To ensure that the commercials overall evaluation did not account for possible differences in risk behavior, we checked whether participants’ evaluations differed. A *t*-test revealed that participants’ evaluation of the neutral commercial (*M* = 4.10, *SD* = 1.01) did not differ from the risk-glorifying commercial (*M* = 4.28, *SD* = 0.95), *t*(50) = -0.65, *p* = .516, *d* = -0.18.

Given that walking speed is affected by gender [63,64], we tested whether participants’ gender was equally distributed across conditions. A chi-square test indicated no difference concerning participants’ gender distribution across conditions, χ^2^ (1, *N* = 51) = 1.70, *p* = .192.

All participants indicated that they were confident that they would participate in two independent studies. However, eight participants did not assume that they actually would have jump off the ramp. Thus, we excluded them from our final sample. Another participant had to be excluded because he or she had a torn ligament. Therefore, 43 participants remained in our final sample. All following computations are based on the remaining 43 participants (*n*_neutral_ = 18, *n*_risk-glorifying_ = 25). Note that all analyses for the total sample are available on the OSF platform (osf.io/rq6yj).

#### Effects of risk-glorifying commercial on risk behavior

**Fig 3:**
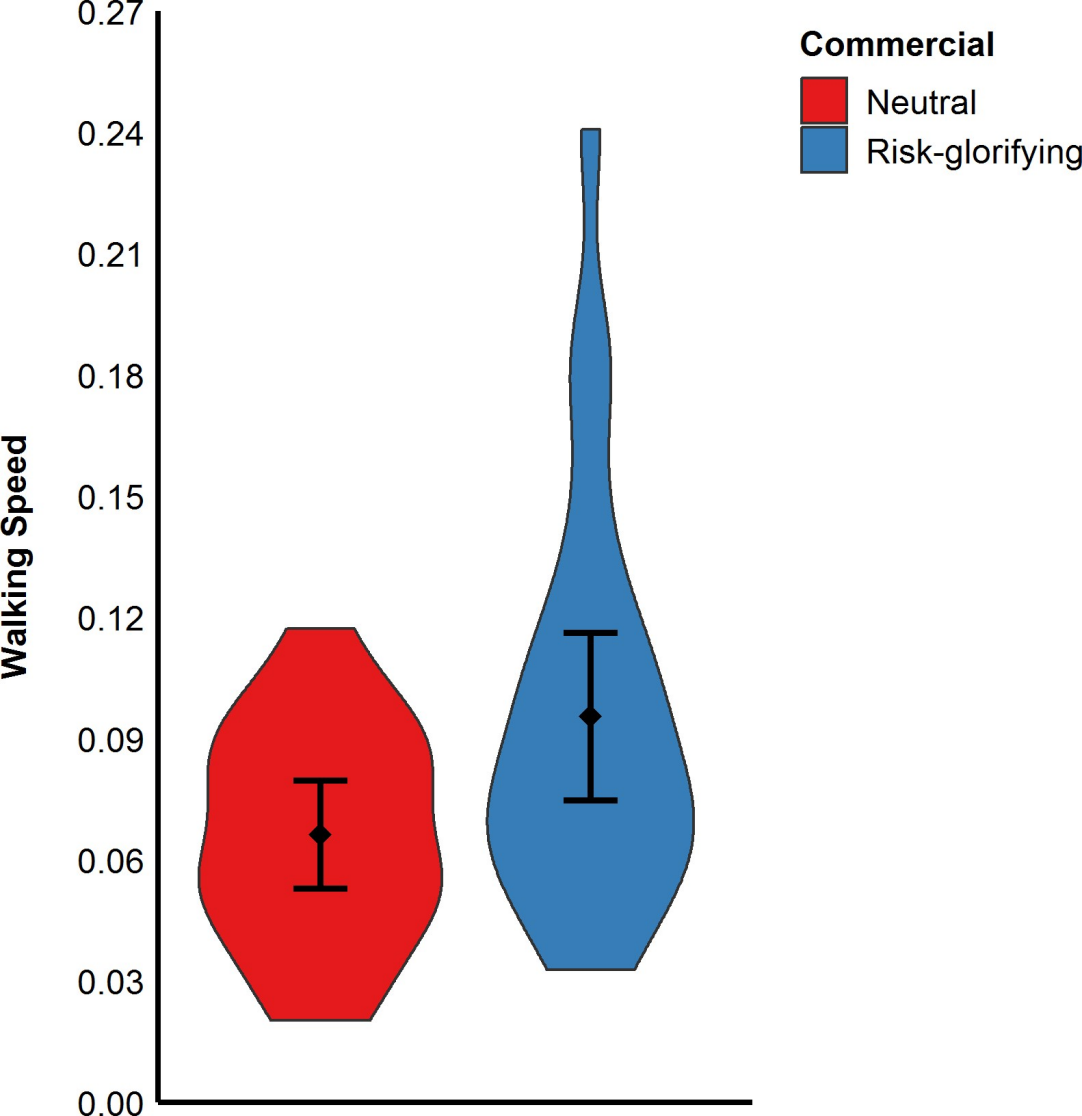
Participants’ walking speed in m/s (Study 1). Error bars represent 95% confidence intervals.

A *t*-test revealed that participants who had been exposed to the neutral commercial walked significantly slower up to the level from which they wanted to jump (*M* = 0.07 m/s, *SD* = 0.03) than those who had been exposed to the risk-commercial (*M* = 0.10 m/s, *SD* = 0.05), *t*(40) = -2.46, *p* = .018, *d* = - 0.69 (see Fig 3).

**Fig 4:**
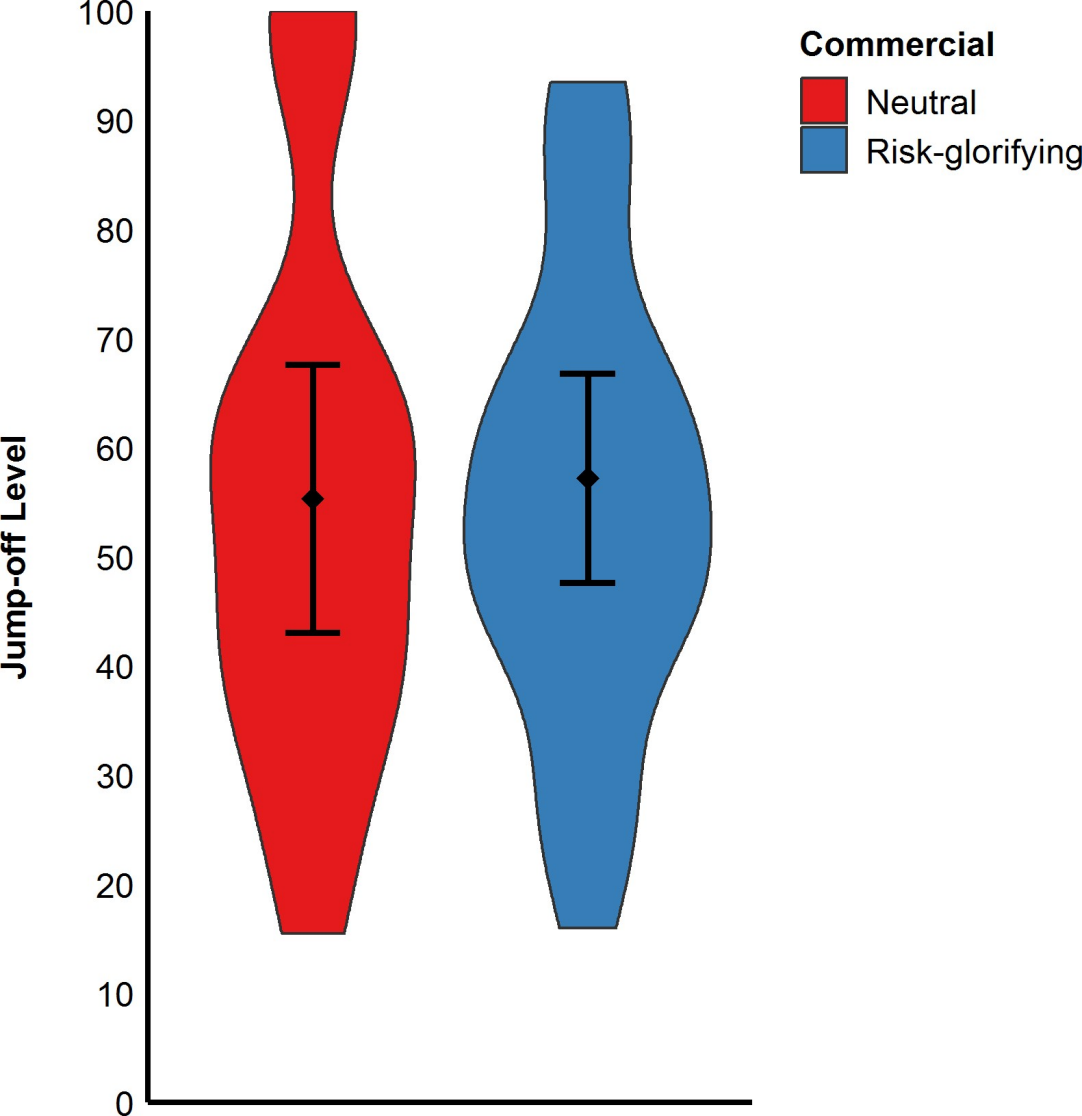
Participants’ jump-off levels in cm (Study 1). Error bars represent 95% confidence intervals.

However, participants in this study did not significantly differ in their level from which they wanted to jump off the ramp. In other words, participants who watched the neutral commercial (*M* = 55.36, *SD* = 24.68) would have jumped off at a similar level as those who watched the risk-glorifying commercial (*M* = 57.23, *SD* = 23.20), *t*(41) = -0.25, *p* = .803. See also Fig 4.

### Discussion

The results of Study 1 reveal that people who had watched risk-glorifying commercials walked faster to the point where they would jump blindfolded off the RAR. However, jump off level was not significantly affected by exposure to risk-glorifying content. Thus, this partially supports the hypothesis that watching a risk-glorifying commercial enhances risk behavior compared to watching neutral commercials.

Although the results of Study 1 show that risk-glorifying commercials foster risk behavior, generalization of these findings may be problematic for two reasons. First, previous research revealed that risk behavior follows an inverted U-function across the human life span, beginning in young adolescence, peaking in the teenage and young adult years, and decreasing in adulthood [65]. Especially, younger people between the ages of 16 and 24 tend have higher risk taking inclinations than people who are older than 24 years [3]. Given that participants’ mean age was below 24 years, the generalizability of Study 1 has to be limited in this way. Second, previous research showed that men behave riskier than women [3]. As participants’ gender ratio was unbalanced in Study 1 (90% females of the total sample), generalization to both genders is limited. Thus, Study 2 aims to enhance generalizability across age and gender.

Moreover, there is evidence that people’s risk behavior is affected by their arousal (for a definition see [66]) levels. For example, people with an increased arousal level are more likely to have unprotected sex [1,67]. Therefore, Study 2 aims to investigate whether additional arousal has a potential mediating effect on risk behavior.

## Study 2

Study 2 aimed to replicate Study 1’s findings and to increase their generalizability by testing a more heterogeneous sample across age and gender as previous research showed that men behave more risky than women [68] and that younger people (i.e., younger than 24 years) behave more risky than older people [3]. Therefore, we attempted to recruit a sample that was balanced concerning participants’ gender and with a mean age above 24 years.

In line with previous research, Study 2 also investigates the potential mediating effect of arousal [3]. However, there are inconsistent findings about the mediating effect of arousal [25,26]. Although the classical approach to test a mediation relies on the measurement of the mediating variable, we tested the mediation following the recommendations of testing mediation by interaction [69]. This approach to mediation allows a comparison of the causal effect observed when the process is uninterrupted to the effect observed when the process is interrupted. Consequently, we directly manipulated arousal to examine it as a potential mediation process between risk-glorifying commercials and actual risk behavior.

As in Study 1, we hypothesize that people who are watching risk-glorifying commercials show increased risk behavior. Additionally, we hypothesize that an increased arousal level increases people’s risk behavior.

### Method

#### Power analysis, participants, exclusion criteria, and design

We based our power analysis on previous research that revealed large effects (Cohen’s *d*’s > 0.8; cf. [17]). To achieve sufficient statistical power (> .80) to detect large effects [51], we aimed for data from 90 participants. We factually collected data from 96 participants (57 female) with an average age of *M* = 34.78, *SD* = 13.70 and a range from 18 to 61 years. They were recruited via email lists and leaflets. In exchange for their participation, participants received a cup of tea or coffee, and chocolate bars. We excluded participants who were not convinced that they would have to jump-off the RAR.

This study was based on a 2 (type of commercial: “neutral” vs. “risk-glorifying”) by 2 (arousal: “additional arousal” vs. “no arousal”) factorial between-subjects design. Our main dependent variable (i.e., risk behavior) was assessed by walking speed and jump-off height. Participants were randomly assigned to one of the four experimental conditions.

#### Procedure and dependent variables

The procedure was almost identical to the procedure in Study 1. In addition, participants had to either do 15 jumping jacks (additional arousal via an increased heart-rate) or to wait for thirty seconds (no arousal) after they had been exposed to a commercial. Jumping jacks have successfully been used to induce arousal (i.e., via increased heart rate) [70–73]. As participants’ evaluation of the commercials did not differ in Study 1, we excluded this measure in Study 2.

#### Check for interfering effects and suspicion

All participants indicated to be confident that they would participate in two independent studies. As in Study 1, we did not further ask for risk perception of the commercials because we did not want to draw participants’ attention to risk behavior to maintain our cover story.

Participants were equally distributed across conditions regarding their age (type of commercial: *F*(1,92) = 3.29, *p* = .073, ω^2^ = 0.02; arousal level: *F*(1,92) = 0.09, *p* = .767, ω^2^ = 0.01; and no interaction between type of commercial and arousal level could be observed: *F*(1,92) = 0.02, *p* = .888, ω^2^ = 0.01. Additionally, a log-linear analysis was performed to check whether participants were equally distributed across conditions regarding their gender. The model indicated no difference concerning participants’ gender distribution across conditions, χ^2^ (4) = 1.19, *p* = .879.

We excluded three participants who did not believe that they had to jump. Therefore, the final sample consisted of 93 participants (risk-glorifying and additional arousal *n* = 23, risk-glorifying and no arousal *n* = 23, neutral and additional arousal *n* = 25, neutral and no arousal *n* = 23). Note that all analyses for the total sample are available on the OSF platform (osf .io/rq6yj).

#### Effects of risk-glorifying commercial and additional arousal on risk behavior

**Fig 5:**
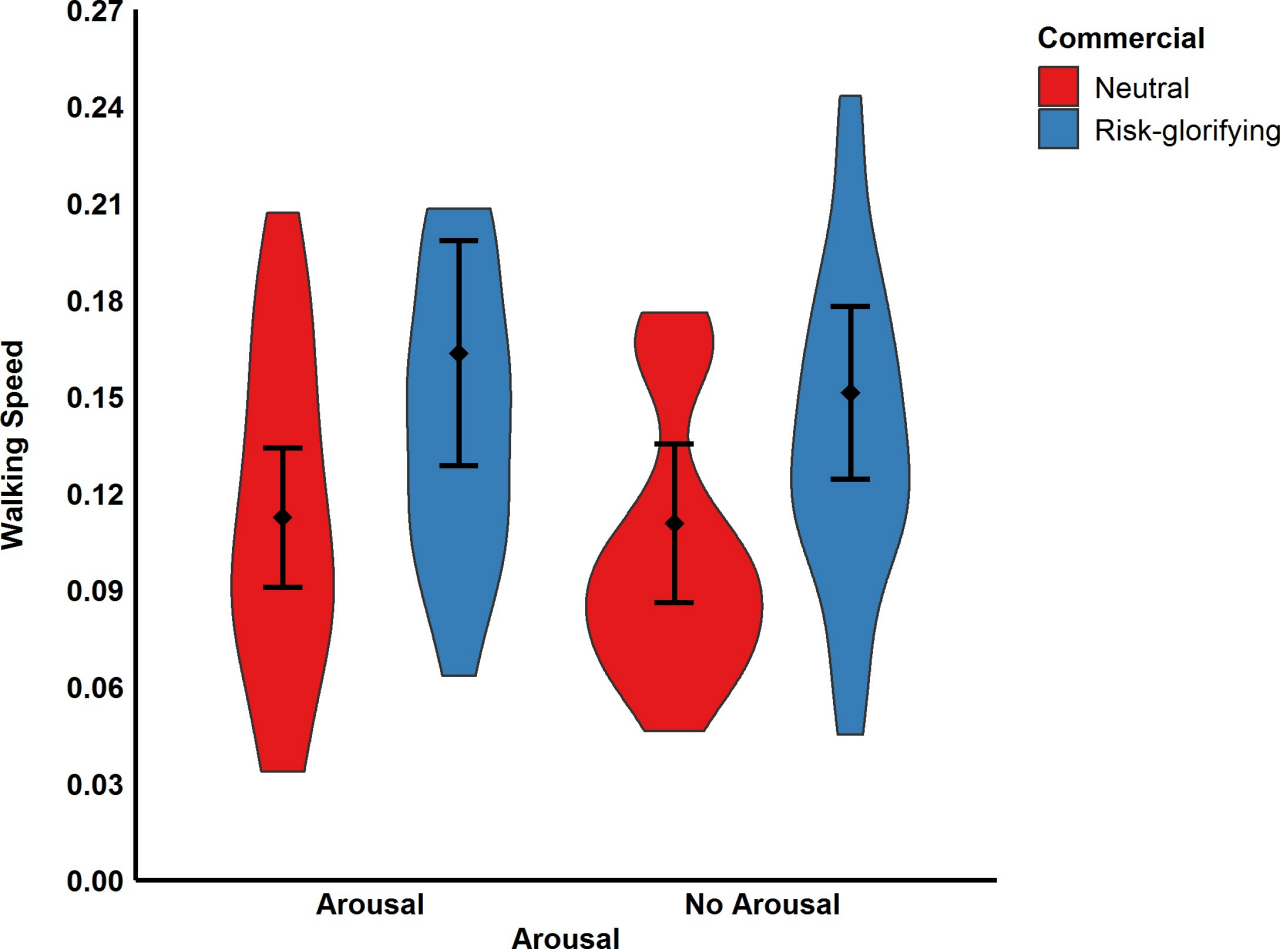
Participants’ walking speed in m/s (Study 2). Error bars represent 95% confidence intervals.

An ANOVA revealed that participants who had been exposed to the risk-glorifying commercial walked significantly faster to the level where they wanted to jump (*M* = 0.16 m/s, *SD* = 0.07) than those who had been exposed to the neutral commercial (*M* = 0.11 m/s, *SD* = 0.05), *F*(1,89) = 12.18, *p* < .001, ω^2^ = .11 (see Fig 5). However, the same ANOVA revealed neither a significant effect for arousal, *F*(1,89) = 0.29, *p* = .591, ω < .01 nor a significant interaction effect, *F*(1,89) = 0.16, *p* = .691, ω^2^ < .01.

**Fig 6:**
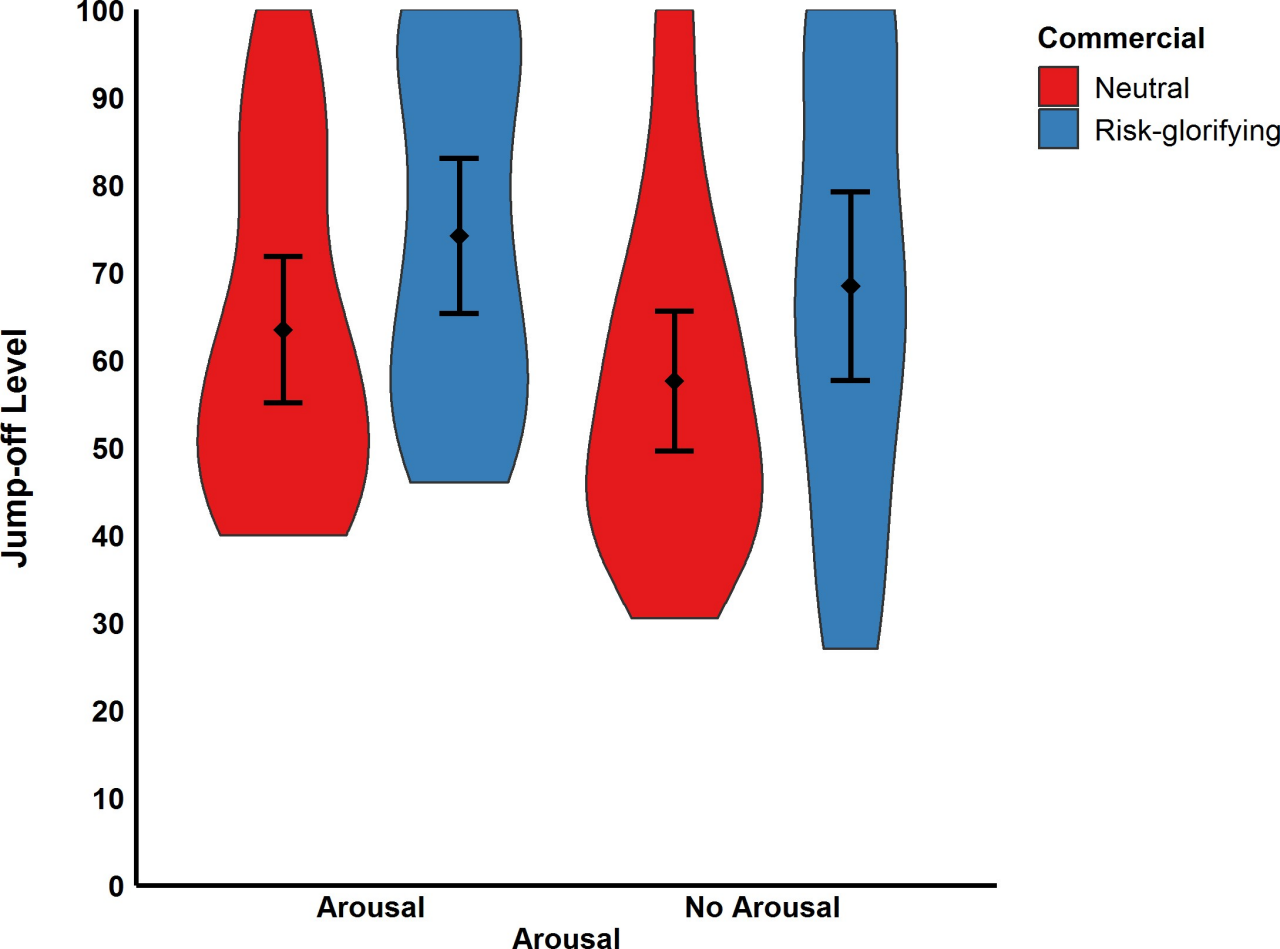
Participants’ jump-off levels in cm (Study 2). Error bars represent 95% confidence intervals.

Moreover, participants in the risk-glorifying condition chose a significantly higher level (*M* = 71.27, *SD* = 22.53) from which they would have been willing to jump-off (see Fig 6) the RAR than those in neutral condition (*M* = 60.43, *SD* = 19.37), *F*(1,89) = 6.12, *p* = .015, ω^2^ = .05. However, the same ANOVA revealed neither a significant effect for arousal, *F*(1,89) = 1.77, *p* = .186, ω^2^ < .01, nor a significant interaction effect, *F*(1,89) = 0.01, *p* = .990, ω^2^ < .01. These results thus indicate that arousal did not mediate the effect of risk-glorifying commercials on risk behavior.

To check whether our findings were affected by participants’ age, we computed a 2 (type of commercial: “risk-glorifying” vs. “neutral”) by 2 (arousal: “no arousal” vs. “arousal”) ANCOVA with age as a continuous control variable. This ANCOVA revealed a significant main effect for the type of commercials, *F*(1,88) = 11.71, *p* < .001, ω^2^ = .11. Participants who were exposed to the risk-glorifying commercial walked faster to the point from which they wanted to jump off the ramp (*M* = 0.11 m/s, *SD* = 0.05) than people who were exposed to the neutral commercial (*M* = 0.16 m/s, *SD* = 0.07). However, there was neither a main effect for arousal, *F*(1,88) = 0.29, *p* = .593, ω^2^ < .01, nor a main effect for age, *F*(1,88) = 0.01, *p* = .981, ω^2^ < .01. Additionally, there was no interaction between type of commercial and arousal, *F*(1,88) = 0.16, *p* = .693, ω^2^ < .01.

Given previous findings that revealed that younger people (i.e., younger than 24) behave riskier than older people do [3], we conducted a 2 (type of commercial: “risk-glorifying” vs. “neutral”) by 2 (arousal: “no arousal” vs. “arousal”) by 2 (age: “younger” vs. “older”) ANOVA. Participants younger than 24 years were coded as “younger”, and those older than 24 as “older”. This ANOVA revealed a significant main effect for the type of commercial, *F*(1,85) = 11.98, *p* < .001, ω^2^ = .11. Participants who were exposed to the risk-glorifying commercial walked faster to the point from which they wanted to jump off the ramp (*M* = 0.11 m/s, *SD* = 0.05) than people who were exposed to the neutral commercial (*M* = 0.16 m/s, *SD* = 0.07). However, there was neither a main effect for arousal, *F*(1,85) = 0.35, *p* = .556, ω^2^ < .01, nor a main effect for age, *F*(1,85) = 0.01, *p* = .954, ω^2^ < .01. Additionally, the ANOVA revealed neither a type of commercial by arousal interaction, *F*(1,85) = 0.21, *p* = .647, ω^2^ < .01, nor other interactions such as type of commercial by age interaction, *F*(1,85) = 0.22, *p* = .640, ω^2^ < .01, arousal by age interaction, *F*(1,85) = 1.01, *p* = .319, ω^2^ < .01, and type of commercial by arousal by age interaction *F*(1,85) = 1.09, *p* = .300, ω^2^ < .01.

These findings were replicated for participants’ jump off levels. An ANCOVA revealed a significant main effect for the type of commercial, *F*(1,88) = 4.98, *p* = .028, ω^2^ = .04. Participants who were exposed to the risk-glorifying commercial would have jumped from a higher level (*M* = 71.27, *SD* = 22.53) than people who were exposed to the neutral commercial (*M* = 60.43, *SD* = 19.37). However, there were no main effects for arousal, *F*(1,88) = 1.72, *p* = .193, ω^2^ < .01, or for age, *F*(1,88) = 2.22, *p* = .140, ω^2^ = .01. Additionally, there was no interaction between type of commercial and arousal, *F*(1,88) = 0.01, *p* = .978, ω^2^ < .01.

The additional ANOVA for comparing younger with older participants revealed a significant main effect for the type of commercial, *F*(1,85) = 5.93, *p* = .017, ω^2^ = .05. Participants who were exposed to the risk-glorifying commercial would have jumped from a higher level (*M* = 71.27, *SD* = 22.53) than people who were exposed to the neutral commercial (*M* = 60.43, *SD* = 19.37). However, the ANOVA revealed neither a main effect for arousal, *F*(1,85) = 0.77, *p* = .381, ω^2^ < .01, nor a main effect for age, *F*(1,85) = 1.27, *p* = .263, ω^2^ < .01. Additionally, there was no interaction between type of commercial and arousal, *F*(1,85) = 0.25, *p* = .618, ω^2^ < .01, and no other interactions such as type of commercial by age interaction, *F*(1,85) = 0.88, *p* = .351, ω^2^ < .01, arousal by age interaction, *F*(1,85) = 1.30, *p* = .257, ω^2^ < .01, and type of commercial by arousal by age interaction, *F*(1,85) = 2.81, *p* = . 097, ω^2^ = .02.

Finally, to check whether our findings were affected by participants’ gender, we computed a 2 (type of commercial: “risk-glorifying” vs. “neutral”) by 2 (arousal: “no arousal” vs. “arousal”) by 2 (gender: “females” vs. “males”) ANOVA. For participants’ walking speed, an ANOVA revealed a significant main effect for the type of commercials, *F*(1,85) = 11.95, *p* < .001, ω^2^ = .10. Participants who were exposed to the risk-glorifying commercial walked faster to the point from which they wanted to jump off the ramp (*M* = 0.16 m/s, *SD* = 0.07) than people who were exposed to the neutral commercial (*M* = 0.11 m/s, *SD* = 0.05). The same ANOVA also revealed a significant main effect for gender, *F*(1,85) = 7.16, *p* = .009, ω^2^ = .06. Men walked faster (*M* = 0.16 m/s, *SD* = 0.07) to the point from which they wanted to jump off the ramp than did women (*M* = 0.12 m/s, *SD* = 0.04). However, there was no main effect for arousal, *F*(1,85) = 0.08, *p* = 780, ω^2^ < .01, no type of commercial by arousal interaction, *F*(1,85) = 0.20, *p* = .659, ω^2^ < .01, and no other interactions such as type of commercial by gender interaction, *F*(1,85) = 0.84, *p* = .362, ω^2^ <. 01, arousal by gender interaction, *F*(1,85) = 0.97, *p* = .480, ω^2^ < .01, and type of commercial by arousal by gender interaction, *F*(1,85) = 0.75, *p* = .390, ω^2^ < .01.

These findings were replicated for participants’ jump-off levels. An ANOVA revealed a significant main effect for the type of commercial, *F*(1,85) = 4.81, *p* = .031, ω^2^ = .04. Participants who were exposed to the risk-glorifying commercial would have jumped off from a higher level (*M* = 71.22, *SD* = 22.53) than people who were exposed to the neutral commercial (*M* = 60.43, *SD* = 19.37). The same ANOVA also revealed a significant main effect for gender, *F*(1,85) = 6.34, *p* = .014, ω ^2^ = .05. Men would have jumped off from a higher level (*M* = 72.65, *SD* = 21.77) than women (*M* = 60.63, *SD* = 20.10). However, there was no main effect for arousal, *F*(1,85) = 1.55, *p* = 780, ω^2^ = .06, no type of commercial by arousal interaction, *F*(1,85) = 0.10, *p* = .948, ω^2^ < .01, and no other interactions such as type of commercial by gender interaction, *F*(1,85) = 0.09, *p* = .759, ω^2^ <. 01, arousal by gender interaction, *F*(1,85) = 0.07, *p* = .795, ω^2^ < .01, and type of commercial by arousal by gender interaction, *F*(1,85) = 0.19, *p* = .668, ω^2^ < .01.

Taken together, our results indicate that people who were exposed to the risk-glorifying commercial behaved riskier than those who were exposed to the neutral commercial. Arousal did not mediate the effect of risk-glorifying commercials on risk behavior. Finally, men behaved riskier than women did, however, this result did not modulate or interact with the overall effect of risk-glorifying commercials on risk behavior.

### Discussion

In line with Study 1, the results revealed that people who had watched risk-glorifying commercials behaved riskier than those who had watched a neutral commercial. Particularly, watching a risk-glorifying commercial increased participants’ walking speed to the point from which they would have jumped blindfolded off the RAR. Additionally, Study 2 shows that people who had watched a risk-glorifying commercial were willing to jump blindfolded off the RAR at a higher level than people who had watched a neutral commercial.

Study 2 extends the generalizability of Study 1’s findings in two aspects, namely, age and gender. First, previous research showed that younger people (i.e., younger than 24 years) are more affected by risk-glorifying media than older people [3,65]. Therefore, we tested an older and more age heterogeneous sample. Thus, we argue that our results can be generalized concerning people’s age. Second, previous research showed that men behave slightly riskier than females [3]. Therefore, we tested a gender-balanced sample. Thus, we argue that our results can be generalized concerning people’s gender.

Study 2 also extends the findings of Study 1 by investigating arousal as an underlying psychological process. Based on previous findings we assumed that increased arousal should drive the effect of risk-glorifying media content on risk behavior. Although, we directly manipulated arousal by using an established method to induce arousal [70,71], our results showed no mediating effect of arousal [69], indicating that an increased arousal level did not increase participants’ risk behavior.

## Study 3

Study 3 aimed to replicate and confirm the results of Study 1 and Study 2 that exposure to risk-glorifying commercials increases risk behavior. Additionally, Study 3 examined whether the effect is mediated by risk-positive cognition. Based on previous findings, we assume that the exposure of risk- glorifying commercials has an impact on the accessibility to risk-related cognitions, which, in turn, may increase risk behavior [3,17,25,33]. Accordingly, we hypothesize that risk- glorifying commercials increase accessibility to risk-positive cognitions that foster individuals’ risk behavior.

### Method

#### Power analysis, participants, exclusion criteria, and design

We based our power analysis on previous research that revealed large effects (Cohen’s *d*’s > 0.8; cf. [17]). To achieve sufficient statistical power (> .80) to detect large effects [51], we aimed for data from 90 participants. We factually collected data from 94 participants (69 female) with an average age of *M* = 21.53, *SD* = 2.60 and a range from 18 to 32 years. Participants received course credit in exchange for their participation. Participants were recruited via postings on the hallways at the psychology department at a University.

This study was based on a one-factorial (type of commercial: “neutral” vs. “risk-positive”) between-subjects design. We additionally assessed risk-positive cognitions after the exposure to the commercials. As in Studies 1 and 2, our main dependent variable (i.e., risk behavior) was assessed by walking speed and jump-off height. Participants were randomly assigned to one of the two experimental conditions.

#### Procedure and dependent variables

The procedure was almost identical to the procedure in Study 1, except for the assessment of participants’ accessibility to risk-positive cognitions after their exposure to one of the two commercials. In line with previous research, we tested the accessibility to risk-positive cognitions via a homonymous decision task [25,26]. Participants received a list of 18 words in which each word had two possible meanings (homonym). Their task was to define the words. Nine words were neutral homonyms—neutral words, which could have two possible meanings, but no risk related meanings. Neutral words were included, because we did not want to draw participants’ attention to risk behavior to maintain our cover story. The other nine words were homonyms with both a neutral and a risk-related meaning. For example, participants had to decide on the meaning of the German word “*wagen”*, which mean “car” (non-risk-related meaning), as well as “to risk something” (risk-related meaning). All homonyms were presented in random order. The absolute number of risk-related word definitions was used as a measure of cognitive accessibility of risk-positive cognitions.

### Results

#### Check for suspicion and interfering effects

By the end of the experiment, all participants were confident that they would participate in two independent studies. A *t*-test revealed no significant difference regarding participants’ age across experimental conditions, *t*(91) = 1.40, *p* = .164. Additionally, a chi-square test indicated no difference concerning participants’ gender distribution across conditions, χ^2^ (1, *N* = 94) = 0.33, *p* = .564. We excluded three participants who did not believe that they had to jump. Therefore, the final sample consisted of 91 participants (*n*_neutral_ = 45, *n*_risk-positive_ = 46). Note that all analyses for the total sample are available on the OSF platform (osf.io/rq6yj).

#### Effects of risk-positive commercial on risk behavior and accessibility of risk-positive cognitions

**Fig 7:**
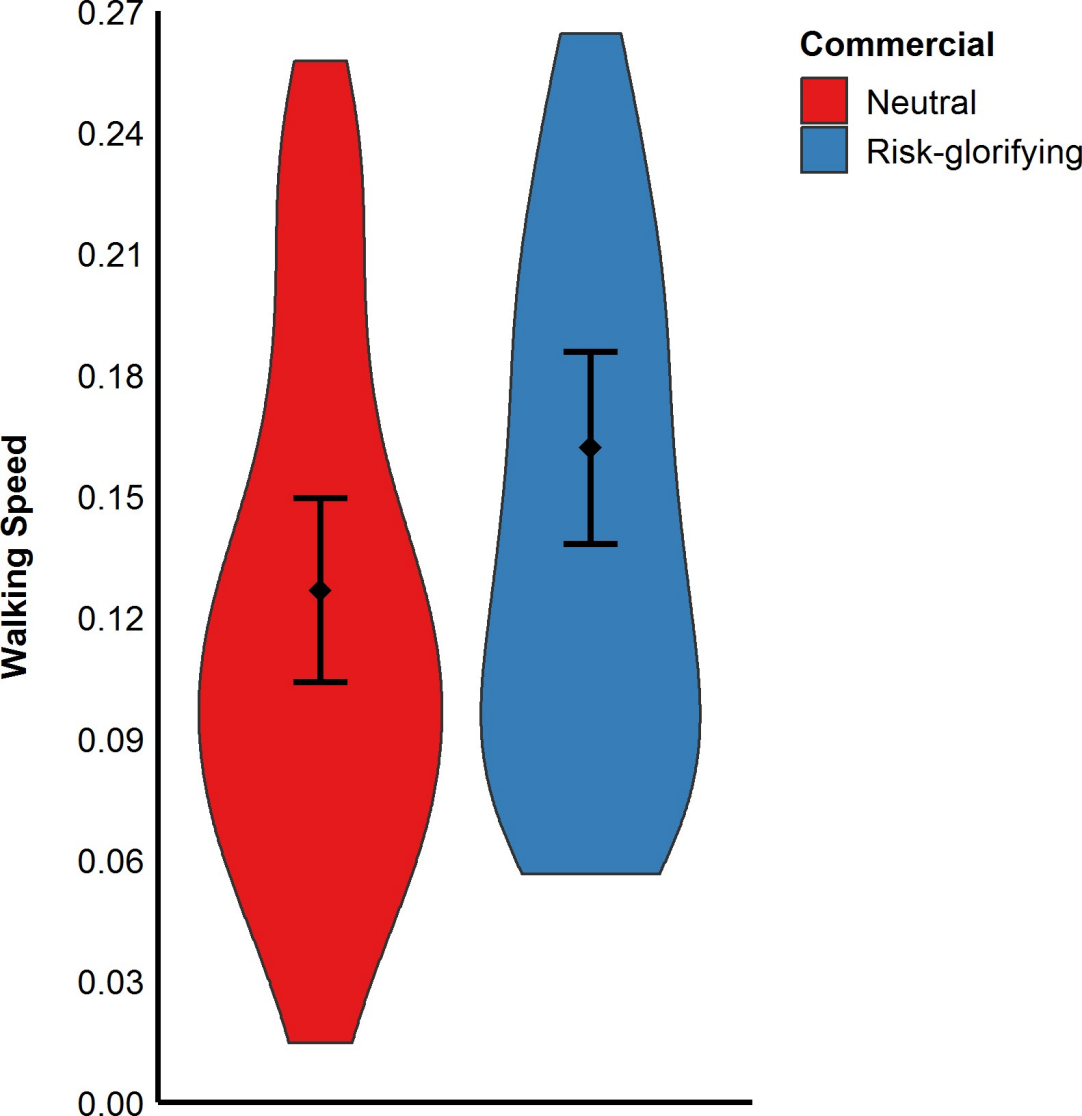
Participants’ walking speed in m/s (Study 3). Error bars represent 95% confidence intervals.

**Fig 8:**
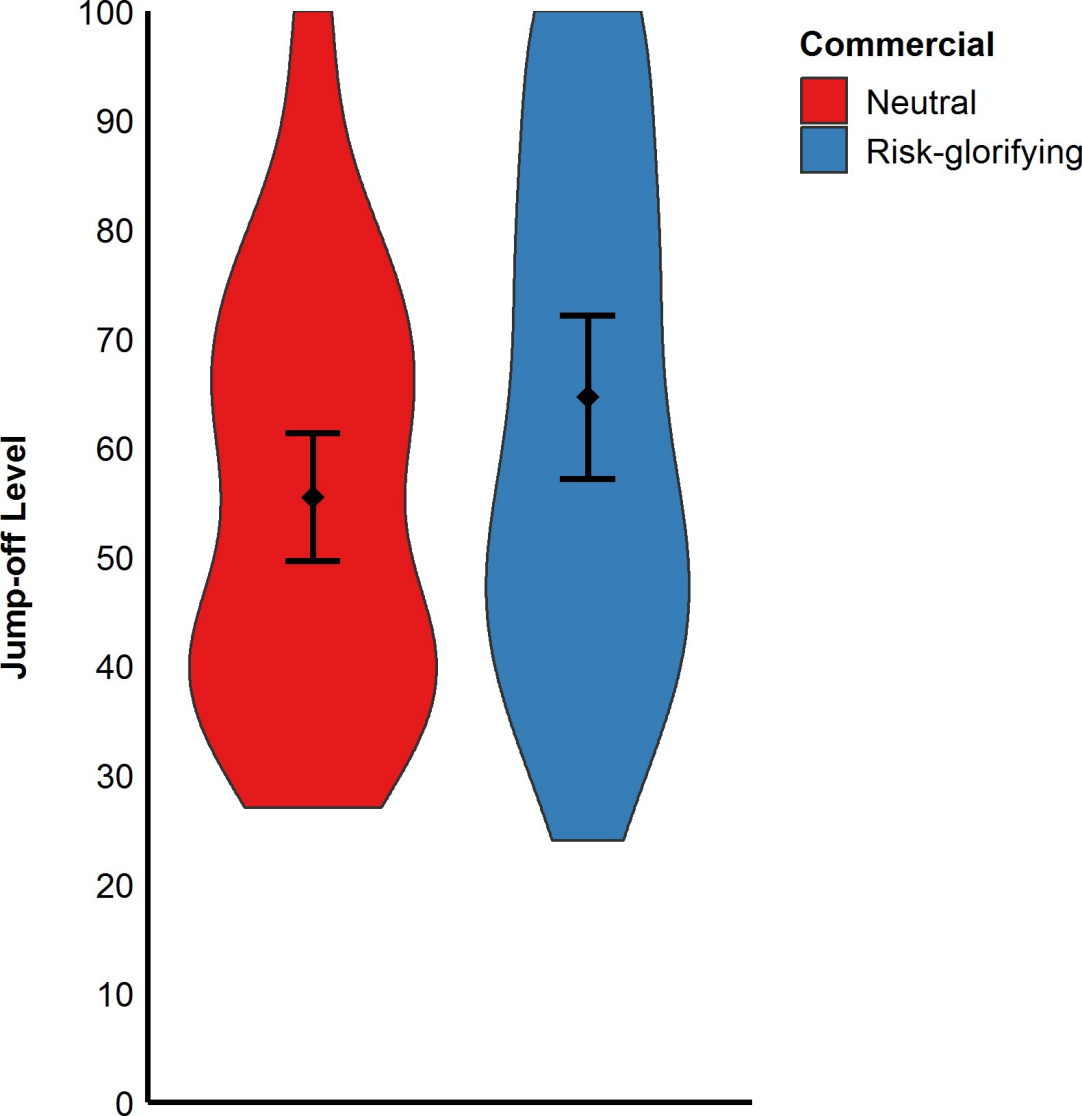
Participants’ jump-off levels in cm (Study 3). Error bars represent 95% confidence intervals.

As in the previous studies, participants exposed to the risk- glorifying commercial walked significantly faster to the level from which they wanted to jump off (*M* = 0.16 m/s, *SD* = 0.08) than those who had been exposed to the neutral commercial (*M* = 0.13 m/s, *SD* = 0.07), *t*(89) = 2.16, *p* = .033, *d* = 0.46, (see Fig 7). Moreover, participants in the risk- glorifying condition chose a higher level (*M* = 64.65, *SD* = 25.19) from which to jump off the RAR than those in neutral condition (*M* = 55.50, *SD* = 19.60), *t*(89) = 1.93, *p* = .056, *d* = 0.41 (see Fig 8). Participants who had been exposed to the risk-glorifying commercial had an increased accessibility to risk-positive cognitions (*M* = 5.78, *SD* = 1.76) than those in the neutral condition (*M* = 4.29, *SD* = 1.38), *t*(89) = 4.51, *p* < .001, *d* = 0.95.

Given that men, on average, behave riskier than do women [68], we checked whether gender affected our findings. An ANOVA with the factors type of commercial and gender revealed no main effect for gender on walking speed, *F*(1,84) = 1.84, *p* = .179, ω^2^ < .01. Thus, men (*M* = 0.17 m/s, *SD* = 0.10) and women (*M* = 0.14 m/s, *SD* = 0.07) did not significantly differ in their walking speed. While the expected main effect of type of commercial was found, *F*(1,84) = 3.87, *p* = .052, ω^2^ = .03, there was no interaction between gender and type of commercials, *F*(1,84) = 0.20, *p* = .653, ω^2^ < .01.

This finding was echoed by a similar pattern for the jump off level. As expected, there was a significant main effect for the type of commercial, *F*(1,87) = 3.78, *p* = .055, ω^2^ = .13. Men (*M* = 75.65, *SD* = 22.86) jumped off from a higher level than women did (*M* = 54.88, *SD* = 20.60), *F*(1,87) = 15.12, *p* < .001, ω^2^ = .148. There was no interaction between gender and type of commercials, *F*(1,87) = 0.57, *p* = .451, ω^2^ < .01. Thus, gender effects did not moderate the media effect on walking speed and jump off level. Additionally, participants’ gender did not significantly affect the accessibility to risk-positive cognitions, *F*(1,87) = 0.29, *p* = .865, ω^2^ < .01. There was no interaction between gender and type of commercials, *F*(1,87) = 0.02, *p* = .902, ω^2^ < .01.

#### Mediational analysis for the impact of risk-positive cognitions on risk behavior

We tested whether accessibility of risk-positive cognitions mediated the effect of risk- glorifying commercials on actual behavior, as proposed in the literature [3,17,25]. A mediation analysis revealed that participants exposed to the risk- glorifying commercial showed increased accessibility to risk-positive cognitions, *a* = -1.50, *t* = -4.39, *p* < .001, 95% CI [-2.18, -0.82]. However, participants who had an increased accessibility to risk-positive cognitions did not walk faster up the ramp, *b* = 0.01, *t* = 1.09, *p* = .279, 95% CI [-0.01, 0.02]. A bias-corrected bootstrap confidence interval for the indirect effect of accessibility of risk-positive cognitions (*ab* = -0.01) included zero (-0.03 to 0.01). This indicates that accessibility to risk-positive cognitions did not mediate the effect of risk-positive commercials on walking speed.

A second mediation analysis revealed a similar pattern for participants’ jump off levels. Again, exposure to a risk-glorifying commercial increased accessibility to risk-positive cognitions, *a* = -1.50, *t* = -4.39, *p* < .001, 95% CI [-2.18, -0.82], but increased accessibility to risk-positive cognitions did not affect jump off heights, *b* = -1.83, *t* = 1.23, *p* = .223, 95% CI [-4.98, 1.01]. A bias-corrected bootstrap confidence interval for the indirect effect of accessibility of risk-positive cognitions (*ab* = 2.96) included zero (-2.08 to 9.01). Thus, the accessibility to risk-positive cognitions did not mediate the effect of risk-glorifying commercial on jump-off level.

### Discussion

In line with Studies1 & 2, Study 3 revealed that people who watch risk-glorifying commercials behave more risky than those who watch a neutral commercial. More specifically, participants who watched a risk-glorifying commercial increased their walking speed to the point from which they would jump blindfolded off the RAR and chose a higher level to jump from than people who watch a neutral commercial.

Although the results did not show the potential mediating effect of accessibility to risk-positive cognitions on risk behavior, Study 3 contributes to a better understanding of the underlying mechanisms. In line with previous findings, Study 3 showed that the exposure to risk-positive media content lead to an increased accessibility to risk-positive cognitions [3,17,25,33]. However, the increased accessibility did not lead to increased risk behavior. This finding can be potentially explained by the fact that convergent validity of propensity and behavioral measurements is low [41].

## Internal meta-analysis

**Fig 9:**
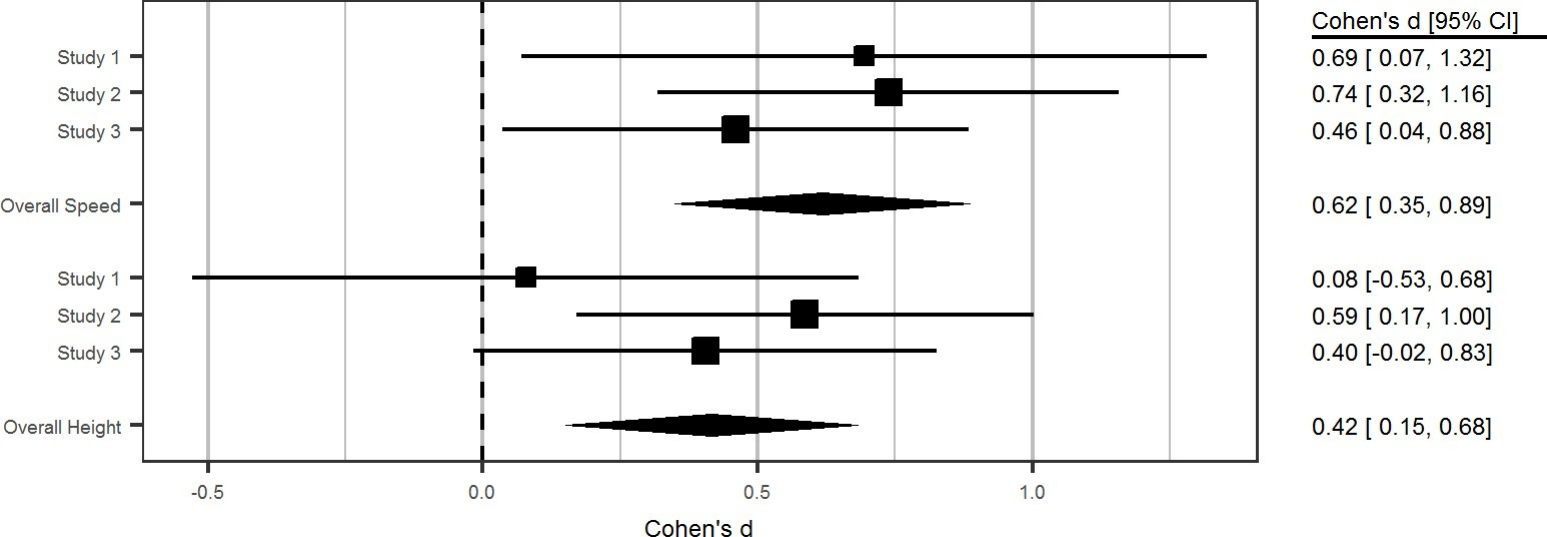
Internal meta-analytic summary of effect sizes.

Given the rather small sample size of Study 1 and the somewhat intermixed effect of the risk-glorifying commercials on participants’ jump-off levels, we meta-analyzed our three studies. We used fixed effects in which the mean effect size was weighted by sample size [74]. We first converted our ω^2^ to η^2^, which we then converted to Cohen’s *d* [51]. The meta-analysis revealed a significant medium to large effect for the impact of risk-glorifying commercials on walking speed (*d* = 0.62, *SE* = 0.14, 95% CI = 0.35–0.89, *Z* = 4.50, *p* < .001). Similarly, the meta-analysis revealed a significant small-to-medium effect for the impact of risk-glorifying commercials on jump-off height (*d* = 0.42, *SE* = 0.14, 95% CI = 0.15–0.68, *Z* = 3.07, *p* < .001). Thus, our findings indicate that the exposure to risk-glorifying commercials increases people’s risk behavior (see Fig 9).

## General discussion

The present research aimed to address the question of whether exposure to risk-glorifying commercials causally leads to increased risk behavior. In line with our assumptions, our results reveal that people who watch risk-glorifying commercials behave riskier than people who watch neutral commercials. Viewing a risk-glorifying commercial led to an increased pace while walking to the jump-off level of the RAR (Studies 1, 2, & 3). Additionally, people who watched a risk-glorifying commercial would jump-off at a higher level of the RAR (Study 2 & Study 3). These findings are strengthened by the results of the internal meta-analysis. Thus, our findings indicate that the exposure to risk-glorifying media contents (i.e., commercials) can foster actual risk behavior.

Although previous research has provided great insight in the relation between risk-glorifying media content and risk behavior, to the best of our knowledge, our findings show a causal link between the exposure to risk-glorifying commercials and risk behavior (i.e., walking speed and jump-off level) for the first time. Therefore, our findings add some important insights regarding the relation between risk-glorifying media content and risk behavior.

First, our findings extend previous experimental research by assessing risk behavior. This is important because previous experimental research mainly focused on effects of risk-glorifying media content on risk-positive emotions and cognitions [17,32,33], or assessed risk-taking inclinations in simulated (i.e., road traffic) situations [16,26] (for a notable exception regarding the impact of risk-glorifying commercials on alcohol consumption see [7,39]).

Second, our findings extend previous correlational research that assessed risk behavior [29,34–36,38] by showing a causal link between risk-glorifying media content and risk behavior. Based on the predictions of socio-cognitive models of learning through experience or observation [42,43] our findings contribute to a better understanding of the question of why people may copy risk behavior (i.e., stunts) that they had seen before in risk-glorifying media (i.e., commercials). Given that our dependent variables assess speed and height, we argue that our findings should generalize to other types of risk behavior that include physical risks caused by height and speed. Nevertheless, we are aware that our findings may not generalize to other risk domains such as gambling, alcohol consumption, or unprotected sexual intercourse. For an elaboration on this point, see limitations and future directions below and [41,75,76].

Third, from a theoretical point of view, the present research contributes to a better understanding of the underlying psychological processes (i.e. arousal). The results of Study 2 revealed that arousal did not mediate the effect of risk-glorifying media content on risk behavior. People with an increased arousal level did not behave riskier than those in the neutral condition, although we directly manipulated arousal [69]. A potential explanation for the non-mediating effect of arousal is that participants can attribute the arousal to other factors [77]. This offered explanation could potentially help to clarify the inconsistent findings on the mediating effects of arousal in the existing literature [1,25,26,67]. We are aware that the statistical power for detecting the interaction effect was rather small. Nevertheless, our results did not indicate a trend for the mediation effect of arousal, on the contrary, our results indicate a rather negligible effect (i.e., ω^2^ < .01).

Moreover, in line with previous findings, the results of the present research indicate that the exposure to risk-glorifying media content (i.e. commercials) increases the accessibility to risk-positive cognitions [3,17,25,33]. Interestingly, our results show that accessibility to risk-positive cognitions did not increase people’s risk behavior. Again, we are aware of the rather small statistical power for detecting the mediation. Nevertheless, our results did not show a trend for the mediation effect of the accessibility to risk-positive cognitions; they showed a rather negligible effect. However, our findings do not rule out the idea that other factors might mediate the effect of risk-glorifying commercials.

From a theoretical point of view, this finding raises the question of, which psychological process transfers the accessibility of risk-positive cognitions to risk behavior. One potential mediator could be the identification with the actors [32]. For example, people who identify themselves as “thrill-seeking extreme mountain bikers” might behave riskier. Closely related, wishful identification with an actor could be a potential mediator [78]. In contrast to completely identifying with what the portrayed actor does, people have the desire to be like or act like the portrayed actor, which, in turn, leads to increased risk behavior. Another potential mediator could be transportation [79,80]. Transportation means that viewers of risk-glorifying commercials immerse themselves in the portrayed scenario without necessarily identifying with an actor. For example, a person who watches a risk-glorifying commercial can immerse his- or herself into the atmosphere of the cheering crowd of 30,000 people at the Plaza de Toros Monumental in Mexico City and hence behave riskier.

Beyond improving the understanding of how risk-glorifying media content is transferred to risk behavior, the present study also helps to better understand why the convergent validity of propensity and behavioral measurements is relatively low [41]. Finally, the presented risk assessment ramp (RAR) is a new method to assess people’s risk behavior, especially, in experimental settings.

### Limitation and future directions

Although our results revealed evidence for a causal link between the exposure to risk-glorifying commercials and risk behavior, there are few limitations. However, we do believe that these limitations provide potential starting points for fruitful future research. The first limitations is that we used only one neutral and one risk-glorifying commercial. Particularly, the used commercials were rather long compared to average broadcasted commercials, which might have attenuated the effect. However, we argue that people who watch TV do not watch a single commercial. Instead they are exposed to the same commercial several times, which, in turn, increases the total time that they are exposed to a commercial [81]. Therefore, we encourage future research to use several commercials (neutral and risk-glorifying), with varying duration as stimuli to increase external and construct validity [82].

Second, some might argue that the present research may be limited because we did not include a prevention condition (i.e., a commercial that should people prevent from participating in self-harming actions). Additionally, we are aware of the fact that the commercial in the neutral condition is slightly above the risk content scale’s mid-point. As a consequence, future research should examine whether people behave less risky if they watch risk-prevention commercials.

Third, some might argue that the result of Study 1 is to some extent inconsistent with the findings (Study 2 and Study 3) that the exposure to risk-glorifying media content increases participants’ jump off levels. This potential inconsistency can be explained by the lower statistical power of Study 1 compared to the statistical power of Study 2 and Study 3 as we found a descriptive albeit not significant difference in jump off level in Study 1.

Fourth, based on our proposed explanation for the inconsistent findings on the mediating effect of arousal, we encourage future research to test whether participants attribute their arousal to the stimulus material or to external factors [77].

Fifth, our studies revealed that people behave riskier after exposure to a risk-glorifying commercial. However, some might argue that this, relatively short-term, effect may not transfer to long-term effects. Although previous research [26] revealed that players of a risk-promoting video game showed increased levels of risk taking inclinations 24 hours after paying, we encourage future research to examine long-term effects of risk-glorifying media.

Sixth, although our findings help to better understand how and whether or not risk-promoting media content transfers to behavior, we are aware that additional psychological processes could be at work, such as or identification with the actor [32,78]. For example, people could identify themselves as “thrill-seeking extreme mountain bikers” and, hence, behave riskier. Therefore, future research should emphasize on clarifying the underlying psychological processes, especially regarding the transferring mechanism between the accessibility to risk-positive cognitions and risk behavior. A promising approach could be the examination of the moderating effect of cognitions on the perception of emotions, which in turn affect risk-behavior. More precisely, positive media-elicited emotions (e.g., joy) should directly affect (i.e., increase) risk-behavior, whereas, the impact of negative media-elicited emotions (e.g., fear) should be moderated by recipients’ judgment of personal relevance and reality [83,84]. For example, the portrayal of a perfectly landed kiss of death backflip could elicit joy, which in turn should directly affect people’s risk-behavior. Conversely, the portrayal of a badly crashed kiss of death backflip could elicit fear, whose effect on risk-behavior should be moderated by people’s perceived implications for their well-being. When people perceive the portrayal as non-threatening for their well-being the effect of fear on risk-behavior diminishes, whereas not when the portrayal is perceived as threatening.

Although previous research that used a comparable measure revealed satisfying retest-reliabilities (i.e., *r*_*tts*_ = .81–92 [85]), we are aware of that we did not assess RAR’s validity. Consequently, we highly encourage future research to close this gap by assessing RAR’s construct, criterion, predictive, and convergent validity with established risk measures such as the accessibility to risk-positive cognitions and emotions [25], financial decisions (e.g., Sequential Investment Task [86]) and video-simulated critical road traffic situations (i.e., The Vienna Risk-Taking Test–Traffic [87]). Relatedly, we are aware that people’s risk behavior may be context-dependent [41,75,76]. For example, one person might behave risky in financial decisions and, be highly risk aversive in traffic situations, while a second person might show the reverse pattern. Thus, following the Brunswikian idea of systematic and representative research designs [88,89], we highly encourage future research to systematically vary stimuli (e.g., commercials, video games, music, enactments, etc.) and their contents (e.g., commercials for: high risk financial investments, risk-glorifying sports, sexual behavior, etc.; consequences: positive vs negative; and intensity: high vs. low), as well as systematically vary the assessment of risk to further clarify whether risk behavior is domain specific and what domains might be triggered by the same stimuli.

Given that scholars have to balance actual risk behavior with the ethical necessity to ensure participants safety in experimental settings, we nevertheless believe that the RAR is an effective and easy-to-apply tool to assess risk behavior for following reasons. Maximum speed has been successfully used to assess people’s risk-taking behavior (i.e., in the context of driving [17,90]). It has been shown that an increased speed is associated with an increased probability to crash (for a review see [90]) and that crashing at higher speed increases the probability of severe or fatal injuries [91–93]. Although walking faster in an experimental setting may be relatively safe and that potential consequences are less drastic than in a driving context, we argue that people who walk faster up the RAR still have an increased risk of hurting themselves. Walking faster increases the risk of falling off the RAR (independent of the maximum height), because it would take people longer to stop.

Beyond that, people with a reduced vision (our participants were blindfolded) adopt a more cautious walking strategy, which manifests by a decreased walking speed [54,55]. Conversely, this means that an increased walking speed indicates less cautious behavior. This argument is nuanced by the finding that walking (without vision impairment) on inclined surfaces, such as the RAR, decreases gait stability [56], which in turn is an indicator for an increased risk of falling [57]. Furthermore, people who would be willing to jump off an increased level have an increased risk of injuring themselves [61], and jumping off an increased level is a type of risk behavior, in which people often engage [58–60]. Finally, reduced vision increases the risk of falling already on a plane surface [62] and given that, our participants were blindfolded, we thus argue that walking speed and jump-off levels are indicators of risk behavior.

### Conclusion

Given that risk-taking behavior is one of the main causes of lethal injuries among children, adolescents, and young adults [2], it is important to understand whether risk-glorifying commercials foster people’s actual risk behavior, especially, under the assumption that people are exposed to a high number of such commercials in their everyday life [81]. The results of the present research indicate that exposure to risk-glorifying commercials leads to increased risk-behavior.
